# Genomic Characterization of Respiratory Syncytial Virus during 2022–23 Outbreak, Washington, USA

**DOI:** 10.3201/eid2904.221834

**Published:** 2023-04

**Authors:** Stephanie Goya, Jaydee Sereewit, Daniel Pfalmer, Tien V. Nguyen, Shah A.K. Mohamed Bakhash, Elizabeth B. Sobolik, Alexander L. Greninger

**Affiliations:** University of Washington, Seattle, Washington, USA (S. Goya, J. Sereewit, D. Pfalmer, T.V. Nguyen, S.A.K. Mohamed Bakhash, E.B. Sobolik, A.L. Greninger);; Fred Hutchinson Cancer Research Center, Seattle (A.L. Greninger)

**Keywords:** respiratory syncytial virus, human orthopneumovirus, genome, COVID-19, respiratory infections, severe acute respiratory syndrome coronavirus 2, SARS-CoV-2, SARS, coronavirus disease, zoonoses, viruses, coronavirus, evolution, genotype, Washington, United States

## Abstract

We sequenced 54 respiratory syncytial virus (RSV) genomes collected during 2021–22 and 2022–23 outbreaks in Washington, USA, to determine the origin of increased RSV cases. Detected RSV strains have been spreading for >10 years, suggesting a role for diminished population immunity from low RSV exposure during the COVID-19 pandemic.

Annual seasonality of respiratory syncytial virus (RSV) in Washington, USA, has been limited primarily to late autumn and winter (*1*). However, an RSV outbreak was not detected during the 2020–21 season because of the COVID-19 pandemic. After lockdowns were relaxed in the summer of 2021, an early RSV season began in August (Figure, panel A). The 2022–23 outbreak also began earlier, but the number of RSV cases was unexpectedly higher than in 2021, alarming public health authorities and the general community (*2*).

**Figure:**
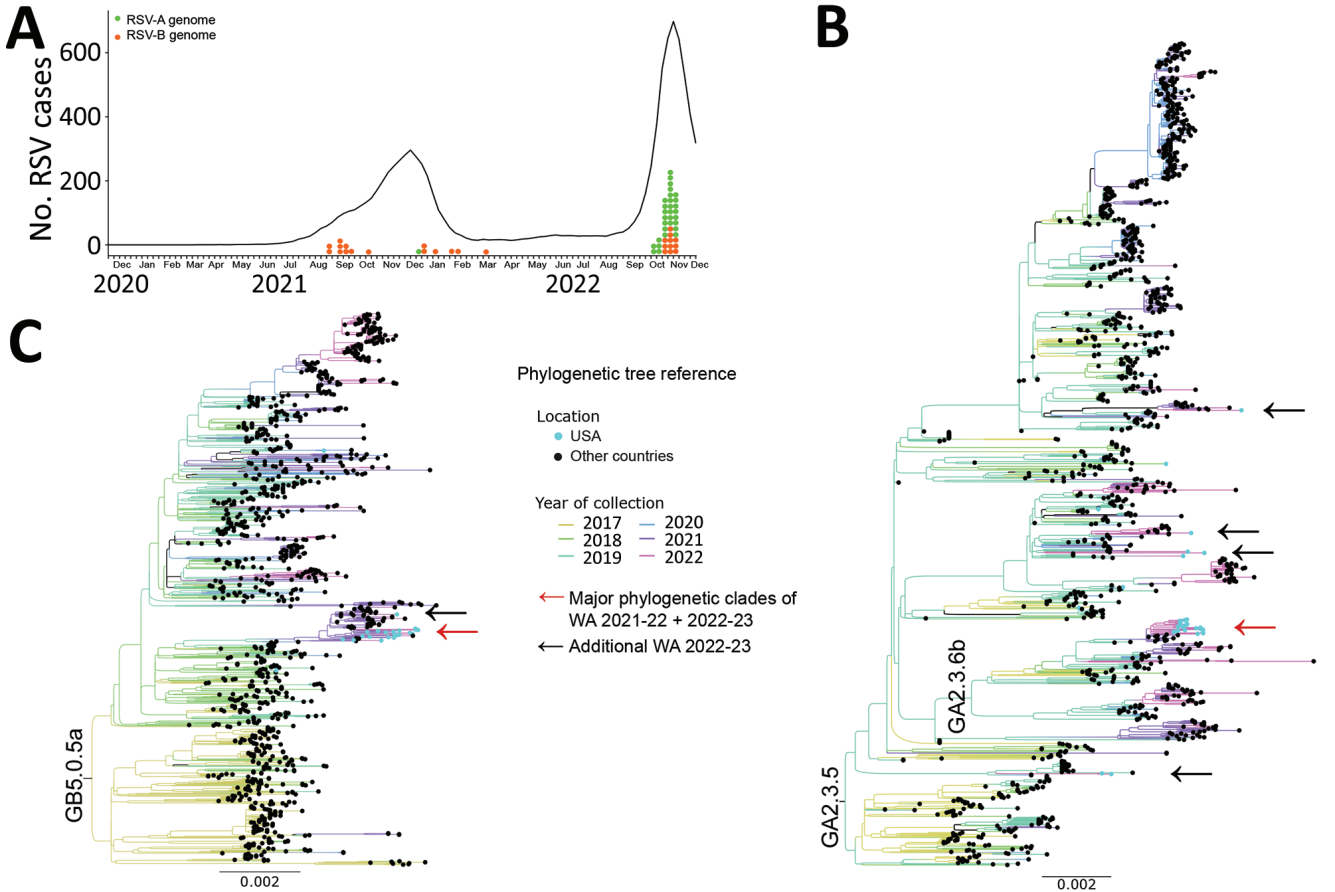
Molecular epidemiology and genomic characterization of RSV during 2021–22 and 2022–23 outbreaks, Washington, USA. A) Number of patients positive for RSV-A and RSV-B during 2021–22 and 2022–23 outbreaks. Graph shows 5-week averages of RSV-positive cases in Washington detected by PCR; data were taken from The National Respiratory and Enteric Virus Surveillance System (https://www.cdc.gov/surveillance/nrevss/index.html) through December 7, 2022. Tick marks indicate weeks for each month beginning on November 28, 2020, and ending on December 3, 2022. Orange and green dots show collection dates for RSV genomes analyzed in this study. B, C) Maximum-likelihood phylogenetic trees of complete genomes of RSV-A (B) and RSV-B (C) collected during 2017–2022. Collection years for specimens are depicted by tree branch color. RSV genomes from the United States are highlighted with light blue circles at branch tips. Red arrow indicates the location of the major phylogenetic clade comprising most of the sequences from Washington during 2021–22 and 2022–23; black arrows indicate locations of other sequences from Washington during 2022–23. Scale bar indicates nucleotide substitutions per site. Complete phylogenetic trees are provided in the Appendix. RSV, respiratory syncytial virus; RSV-A, RSV subtype A; RSV-B, RSV subtype B.

Increased severity of the 2022–23 RSV outbreak might have been caused by diminished protective immunity in the population from prolonged low exposure to this virus (*3*). Furthermore, selective pressure because of low transmission in 2020 might have caused emergence of new viral strains with improved fitness. We evaluated whether RSV causing the 2022–23 outbreak had genomic characteristics different from strains from previous seasons.

We performed hybridization capture-based, metagenomic next-generation sequencing of 54 RSV genomes (14 RSV strains from 2021–22 and 40 from 2022–23) isolated during outbreaks in King County, Washington. In brief, we extracted virus RNA from excess nasal or nasopharyngeal swab specimens collected from persons seeking care at University of Washington Medicine COVID-19 collection sites, clinics, emergency rooms, and inpatient facilities who tested positive for RSV by PCR with a cycle threshold <30 (Table) (*4*). All persons were outpatients except for 2 hospitalized patients from 2021. For phylogenetic analyses, we downloaded complete genomes of RSV-A and RSV-B subtypes from GenBank and GISAID (https://www.gisaid.org) databases. We performed genome alignments by using MAFFT software (https://mafft.cbrc.jp/alignment/software) and constructed phylogenetic trees by using IQ-TREE (*5*) (Appendix). 

**Table:** Number of sequenced respiratory syncytial virus genomes according to different patient characteristics during 2021–22 and 2022–23 virus outbreaks in Washington, USA*

Characteristics	2021–22 outbreak				2022–23 outbreak		
	RSV-A	RSV-B	Total		RSV-A	RSV-B	Total
No. complete genomes	1	13	14		30	10	40
Patient sex							
M	0	6	6		13	7	20
F	1	7	8		16	4	20
Clinical status							
Inpatient	1	1	2		0	0	0
Outpatient	0	12	12		30	10	40
Patient age, y							
<3	0	5	5		10	6	16
3–18	0	3	3		10	2	12
19–65	1	4	5		8	1	9
>65	0	2	2		2	1	3

*RSV, respiratory syncytial virus; RSV-A, RSV subtype A; RSV-B, RSV subtype B.

Among sequenced specimens, we detected 1 RSV-A and 13 RSV-B subtypes from 2021–22 and 30 RSV-A and 10 RSV-B subtypes from 2022–23 (Table). We did not detect co-infections with other respiratory viruses (Appendix) or differences in subtype predominance by patient age group or sex during the 2022–23 outbreak (p>0.1 by Fisher exact test). We genotyped the RSV *G* gene and found that 7 RSV-A sequences were GA2.3.5 and 24 were GA2.3.6b genotypes (both comprising ON1 strains), and all RSV-B sequences were the GB5.0.5.a genotype (BA strains) (*6*) (Appendix). We found that Washington RSV (WA-RSV) sequences were closely related to contemporary viruses by using complete genome phylogenetic analysis with all historical and recent RSV sequences in public databases up to December 2022 (Appendix). We then constructed reduced phylogenetic trees with RSV genomes from public databases collected during 2017–2022 (Figure, panels B, C; Appendix Figures 1, 2); the trees showed the WA-RSV sequences from 2021–22 and 2022–23 outbreaks were closely related to those genomes. However, WA-RSV sequences from 2018 and 2019 were not phylogenetically close to database-derived RSV genomes collected during 2017–2022. Some WA-RSVs from 2022 were individually associated with viruses from France, Spain, Argentina, Brazil, Netherlands, Israel, Australia, and northern Macedonia, isolated in 2019, 2021, or early 2022, suggesting multiple viral introductions within Washington. Nevertheless, most WA-RSVs were within statistically supported monophyletic clades (Figure, panels B, C; Appendix Figures 1, 2), indicating the 2022–23 outbreak in King County has been mainly caused by the same RSV-A and RSV-B lineages observed globally for ≈1 decade. We observed no phylogenetic relationship between clade and patient age.

Analysis of all viral genes from 2022–23 WA-RSVs showed no specific nonsynonymous changes compared with other RSV strains collected globally since 2017. Furthermore, WA-RSVs contained amino acid changes previously identified in sequences isolated before the COVID-19 pandemic. For example, the amino acid constellation A103T and T122A in the RSV-A fusion protein was also detected in 14 other RSV genomes, including a 2019 sequence from the Netherlands (GenBank accession no. MZ515825.1), suggesting a bottleneck effect caused by low transmission during 2020 that reduced virus diversity (*7*). Alternating prevalence of RSV subtypes between outbreaks might also lead to high levels of RSV spread (Table). Further analyses of RSV sequences from Washington and globally are needed to confirm those hypotheses.

The first limitation of our study is that few RSV genomes from Washington were available before the COVID-19 pandemic. Second, we conducted convenience sampling from excess clinical specimens and had limited access to clinical metadata. Nonetheless, Washington is comparatively a well-sampled state for RSV sequences, because only 2 other RSV genomes have been isolated from the rest of the United States since 2017. RSV genomics is also currently limited by a lack of consensus on genotyping classification.

In conclusion, effects of COVID-19 pandemic lockdown measures on the RSV ecosystem have been reported (*8*–*10*). Real-time genomic surveillance of RSV outbreaks in Washington did not reveal specific changes in RSV since the COVID-19 pandemic began that would account for increased viral spread. Our data suggest that RSV reemergence in King County is likely because of diminished protective immunity in the population from low RSV exposure, a consequence of pandemic mitigation measures. With likely future widespread availability of RSV vaccines, continued real-time RSV genomic surveillance will be required to monitor the evolution and emergence of new viral strains.

Appendix Additional information for genomic characterization of respiratory syncytial virus during 2022–23 outbreak, Washington, USA.
