# Supplementary material for: Genomic Characterization of Respiratory Syncytial Virus during 2022–23 Outbreak, Washington, USA
Source: Emerg Infect Dis. 2023 Apr;29(4):865–8. doi: 10.3201/eid2904.221834 (PMC10045680; doi:10.3201/eid2904.221834)
Supplement: Appendix — Additional information for genomic characterization of respiratory syncytial virus during 2022–23 outbreak, Washington, USA. [file 22-1834-Techapp-s1.pdf]

Article DOI: https://doi.org/10.3201/eid2904.221834
EID cannot ensure accessibility for supplementary materials supplied by authors. Readers who have difficulty accessing supplementary content should contact the authors for assistance.

Genomic Characterization of Respiratory Syncytial Virus during 2022–23 Outbreak, Washington, USA

Appendix

Material and methods

RSV genome sequencing

We extracted virus RNA by using the Quick-RNA Viral Kit (Zymo Research, https://www.zymoresearch.com). For samples with RSV PCR cycle threshold counts <25, virus genome sequencing was performed as previously described (*1*). In brief, we used the TURBO DNA-free Kit (ThermoFisher Scientific, https://www.thermofisher.com) to remove genomic DNA from extracted RNA. We performed first-strand cDNA synthesis by using random hexamers and SuperScript IV Reverse Transcriptase (ThermoFisher Scientific) and second-strand synthesis by using Sequenase Version 2.0 DNA Polymerase (ThermoFisher Scientific). We purified double-stranded cDNA by using AMPure XP Magnetic Beads (Beckman Coulter, https://www.beckman.com) before proceeding to tagmentation and library preparation by using the Illumina DNA Prep, (S) Tagmentation kit (Illumina, https://www.illumina.com). For samples with RSV PCR threshold counts of 25–30, we used the RNA Prep with Enrichment, (L) Tagmentation and Respiratory Virus Oligos Panel v2 (Illumina) for library generation. DNA libraries were sequenced as 2 × 100-bp and 2 × 250-bp runs in a NextSeq500 sequencer (Illumina).

Bioinformatic analysis

Quality of FastQ files was analyzed by using FastQC software (http://www.bioinformatics.babraham.ac.uk/projects/fastqc). RSV genome assembly was generated by using the Revica pipeline (https://github.com/greninger-lab/revica). Briefly, adaptor trimming and quality filtering was performed with Trimmomatic v0.39 (*2*). Mapping against a

viral genome reference database was performed, followed by one round of mapping against the virus reference sequence with the highest median coverage (RSV-A reference, GenBank accession no. MZ516076.1; RSV-B reference, GenBank accession no. OK649754.1) and 2 iterations of mapping against the consensus reconstruction. Consensus genomes were generated by using a minimum base quality of 20, minimum depth of coverage of 5 times, and 60% allele frequency. Co-infection in a sample was indicated when 2 complete or partial consensus genomes of different virus species resulted from the Revica analysis.

Comparative analysis of nonsynonymous changes was performed by using consensus genome alignments that included genomes published in GenBank and GISAID (https://www.gisaid.org) with collection dates during January 2017–December 2022 (2,481 total genomes: 1,320 RSV-A and 1,161 RSV-B subtypes). Inclusion criteria comprised complete genomes from clinical isolates with >95% sequence coverage. Alignments were trimmed according to the open reading frame of the virus genes and translated with standard amino acid codes by using Aliview (*3*).

Phylogenetic analysis

RSV genome alignments were built for each RSV subtype by using MAFFT software and visualized with Aliview (*3*,*4*). RSV genotyping was based on the *G* gene (*5*). The sequenced genomes were trimmed to the ectodomain of the *G* gene and analyzed with ReSVidex (https://cacciabue.shinyapps.io/resvidex) and corroborated by maximum-likelihood inference by using RSV-A and RSV-B reference alignments (*5*). *G* gene genotyping trees are available at https://github.com/greninger-lab/RSV-WA-2022. Genotype classification and sequences analyzed in this study are also available on the Nextstrain platform (https://nextstrain.org/rsv/a/G, https://nextstrain.org/rsv/b/G) (*6*).

In accordance with other RSV genotyping schemes, the sequences in this study would be classified as follows: lineage GA2.3.5 would be equivalent to ON1 (*7*), A23 (*8*), A.5.9 (*9*), and NA1 (*10*); lineage GA2.3.6b would be equivalent to ON1 (*7*), A23 (*8*), A.5.11 (*9*), and NA1 (*10*); lineage GB5.0.5a would be equivalent to BA9 (*11*), B6 (*8*), B.5.8 (*9*), and BA (*12*).

For comprehensive phylogenetic analyses, RSV genomes from clinical samples were downloaded from GenBank and GISAID databases if they had <5% N bases. Two phylogenetic analyses were performed: 1 analysis using all available RSV genomes (mentioned in the main

text as historical and recent RSV: 2,195 RSV-A and 1,711 RSV-B) and a reduced analysis that included RSV genomes with collection dates since 2017 (1,320 RSV-A and 1,161 RSV-B). Maximum-likelihood trees were inferred by using IQ-TREE v2.1 (*13*). The molecular evolution model was estimated by using ModelFinder (*14*), and the reliability of sequences clusters was evaluated by using UFBoot2 (10,000 replicates) (*15*). Complete RSV-A and RSV-B tree files and extended versions of the reduced trees (Figure, main text) are available at https://github.com/greninger-lab/RSV-WA-2022.

Data availability

RSV consensus genomes are available in GenBank (accession nos. OP890312–50 and OP965698–712). Sequencing reads are available in NCBI BioProject no. PRJNA907066 (https://www.ncbi.nlm.nih.gov/bioproject). Line-item specimen data are available in the Appendix Table.

GISAID acknowledgment

We thank the authors from the originating laboratories responsible for obtaining the specimens and the submitting laboratories where genetic sequence data were generated and shared via the GISAID Initiative (Authors acknowledgment table available at https://github.com/greninger-lab/RSV-WA-2022).

References

1. Goya S, Valinotto LE, Tittarelli E, Rojo GL, Nabaes Jodar MS, Greninger AL, et al. An optimized methodology for whole genome sequencing of RNA respiratory viruses from nasopharyngeal aspirates. PLoS One. 2018;13:e0199714. PubMed https://doi.org/10.1371/journal.pone.0199714

2. Bolger AM, Lohse M, Usadel B. Trimmomatic: a flexible trimmer for Illumina sequence data. Bioinformatics. 2014;30:2114–20. PubMed https://doi.org/10.1093/bioinformatics/btu170

3. Larsson A. AliView: a fast and lightweight alignment viewer and editor for large datasets. Bioinformatics. 2014;30:3276–8. PubMed https://doi.org/10.1093/bioinformatics/btu531

4. Katoh K, Standley DM. MAFFT multiple sequence alignment software version 7: improvements in performance and usability. Mol Biol Evol. 2013;30:772–80. PubMed https://doi.org/10.1093/molbev/mst010

5. Goya S, Galiano M, Nauwelaers I, Trento A, Openshaw PJ, Mistchenko AS, et al. Toward unified molecular surveillance of RSV: a proposal for genotype definition. Influenza Other Respir Viruses. 2020;14:274–85. PubMed https://doi.org/10.1111/irv.12715

6. Hadfield J, Megill C, Bell SM, Huddleston J, Potter B, Callender C, et al. Nextstrain: real-time tracking of pathogen evolution. Bioinformatics. 2018;34:4121–3. PubMed https://doi.org/10.1093/bioinformatics/bty407

7. Eshaghi A, Duvvuri VR, Lai R, Nadarajah JT, Li A, Patel SN, et al. Genetic variability of human respiratory syncytial virus A strains circulating in Ontario: a novel genotype with a 72 nucleotide G gene duplication. PLoS One. 2012;7:e32807. PubMed https://doi.org/10.1371/journal.pone.0032807

8. Ramaekers K, Rector A, Cuypers L, Lemey P, Keyaerts E, Van Ranst M. Towards a unified classification for human respiratory syncytial virus genotypes. Virus Evol. 2020;6:veaa052. PubMed https://doi.org/10.1093/ve/veaa052

9. Chen J, Qiu X, Avadhanula V, Shepard SS, Kim DK, Hixson J, et al. Novel and extendable genotyping system for human respiratory syncytial virus based on whole-genome sequence analysis. Influenza Other Respir Viruses. 2022;16:492–500. PubMed https://doi.org/10.1111/irv.12936

10. Muñoz-Escalante JC, Comas-García A, Bernal-Silva S, Robles-Espinoza CD, Gómez-Leal G, Noyola DE. Respiratory syncytial virus A genotype classification based on systematic intergenotypic and intragenotypic sequence analysis. Sci Rep. 2019;9:20097. PubMed https://doi.org/10.1038/s41598-019-56552-2

11. Trento A, Viegas M, Galiano M, Videla C, Carballal G, Mistchenko AS, et al. Natural history of human respiratory syncytial virus inferred from phylogenetic analysis of the attachment (G) glycoprotein with a 60-nucleotide duplication. J Virol. 2006;80:975–84. PubMed https://doi.org/10.1128/JVI.80.2.975-984.2006

12. Muñoz-Escalante JC, Comas-García A, Bernal-Silva S, Noyola DE. Respiratory syncytial virus B sequence analysis reveals a novel early genotype. Sci Rep. 2021;11:3452. PubMed https://doi.org/10.1038/s41598-021-83079-2

13. Minh BQ, Schmidt HA, Chernomor O, Schrempf D, Woodhams MD, von Haeseler A, et al. IQ-TREE 2: new models and efficient methods for phylogenetic inference in the genomic era. Mol Biol Evol. 2020;37:1530–4. PubMed https://doi.org/10.1093/molbev/msaa015

14. Kalyaanamoorthy S, Minh BQ, Wong TKF, von Haeseler A, Jermiin LS. ModelFinder: fast model selection for accurate phylogenetic estimates. Nat Methods. 2017;14:587–9. PubMed https://doi.org/10.1038/nmeth.4285

15. Hoang DT, Chernomor O, von Haeseler A, Minh BQ, Vinh LS. UFBoot2: improving the ultrafast bootstrap approximation. Mol Biol Evol. 2018;35:518–22. PubMed https://doi.org/10.1093/molbev/msx281

Appendix Table. Metadata of sequenced RSV specimens*

Sequence name	Collection date	RSV subtype	Ct	Coverage†	Genbank no.	BioProject no.‡	BioSample no.‡	SRA fastq file‡
hRSV/A/USA/202276NDB/2022	10–2022	A	19.20	120.86	OP890312	PRJNA907066	SAMN32118079	SRR22580785
hRSV/A/USA/2022FLDV8/2022	10–2022	A	18.46	87.38	OP890313	PRJNA907066	SAMN32118080	SRR22580784
hRSV/A/USA/2022R3AE2/2022	10–2022	A	18.65	147.96	OP890314	PRJNA907066	SAMN32118081	SRR22580773
hRSV/A/USA/2022AF7QA/2022	10–2022	A	19.08	114.32	OP890315	PRJNA907066	SAMN32118082	SRR22580762
hRSV/A/USA/2022LTGQ4/2022	10–2022	A	19.27	119.10	OP890316	PRJNA907066	SAMN32118083	SRR22580751
hRSV/A/USA/202226672/2022	10–2022	A	20.46	130.82	OP890317	PRJNA907066	SAMN32118084	SRR22580740
hRSV/B/USA/20229B2FA/2022	11–2022	B	19.33	138.62	OP890341	PRJNA907066	SAMN32118085	SRR22580735
hRSV/A/USA/20223TBF2/2022	10–2022	A	19.19	141.67	OP890318	PRJNA907066	SAMN32118086	SRR22580734
hRSV/A/USA/2022TQVY1/2022	11–2022	A	18.78	148.55	OP890319	PRJNA907066	SAMN32118087	SRR22580733
hRSV/A/USA/20229K9JE/2022	11–2022	A	19.78	125.66	OP890320	PRJNA907066	SAMN32118088	SRR22580732
hRSV/A/USA/20222G8K4/2022	10–2022	A	17.13	65.64	OP890321	PRJNA907066	SAMN32118089	SRR22580783
hRSV/A/USA/2022DT79D/2022	10–2022	A	18.84	147.88	OP890322	PRJNA907066	SAMN32118090	SRR22580782
hRSV/B/USA/20229BJQ7/2022	11–2022	B	18.79	126.65	OP890342	PRJNA907066	SAMN32118091	SRR22580781
hRSV/A/USA/202234AM6/2022	11–2022	A	18.82	54.28	OP890323	PRJNA907066	SAMN32118092	SRR22580780
hRSV/A/USA/2022YSFS7/2022	10–2022	A	18.47	53.97	OP890324	PRJNA907066	SAMN32118093	SRR22580779
hRSV/B/USA/2022A7421/2022	10–2022	B	19.68	93.93	OP890343	PRJNA907066	SAMN32118094	SRR22580778
hRSV/A/USA/2022PTEA0/2022	10–2022	A	19.11	123.60	OP890325	PRJNA907066	SAMN32118095	SRR22580777
hRSV/B/USA/2022KH4F2/2022	10–2022	B	19.98	112.33	OP890344	PRJNA907066	SAMN32118096	SRR22580776
hRSV/B/USA/2022RWWYB/2022	10–2022	B	18.43	115.10	OP890345	PRJNA907066	SAMN32118097	SRR22580775
hRSV/A/USA/20227VPLD/2022	10–2022	A	18.83	86.18	OP890326	PRJNA907066	SAMN32118098	SRR22580774
hRSV/A/USA/2022T3TPE/2022	10–2022	A	19.26	85.36	OP890327	PRJNA907066	SAMN32118099	SRR22580772
hRSV/A/USA/2022BARMF/2022	11–2022	A	17.97	126.93	OP890328	PRJNA907066	SAMN32118100	SRR22580771
hRSV/A/USA/20222T321/2022	11–2022	A	17.36	138.17	OP890329	PRJNA907066	SAMN32118101	SRR22580770
hRSV/B/USA/20228BLN9/2022	10–2022	B	20.3	125.53	OP890346	PRJNA907066	SAMN32118102	SRR22580769
hRSV/B/USA/2022RFHA8/2022	11–2022	B	19.59	121.36	OP890347	PRJNA907066	SAMN32118103	SRR22580768
hRSV/A/USA/2022ZFYQ7/2022	11–2022	A	22.53	130.05	OP890330	PRJNA907066	SAMN32118104	SRR22580767
hRSV/A/USA/20226D2SC/2022	10–2022	A	17.85	139.53	OP890331	PRJNA907066	SAMN32118105	SRR22580766
hRSV/A/USA/20228A7L8/2022	10–2022	A	19.74	21.53	OP890332	PRJNA907066	SAMN32118106	SRR22580765
hRSV/A/USA/2022B3ML8/2022	11–2022	A	18.1	10.49	OP965712	PRJNA907066	SAMN32118107	SRR22580764
hRSV/A/USA/2022EH3HA/2022	11–2022	A	20.2	105.85	OP890333	PRJNA907066	SAMN32118108	SRR22580763
hRSV/A/USA/20229BGEF/2022	11–2022	A	20.26	22.35	OP890334	PRJNA907066	SAMN32118109	SRR22580761
hRSV/A/USA/2022EJE74/2022	11–2022	A	17.89	74.50	OP890335	PRJNA907066	SAMN32118110	SRR22580760
hRSV/A/USA/2022F6RQ4/2022	11–2022	A	17.88	101.39	OP890336	PRJNA907066	SAMN32118111	SRR22580759
hRSV/A/USA/20223B286/2022	11–2022	A	19.46	18.19	OP890337	PRJNA907066	SAMN32118112	SRR22580758
hRSV/B/USA/2022YVED3/2022	11–2022	B	19.46	139.35	OP890348	PRJNA907066	SAMN32118113	SRR22580757
hRSV/A/USA/20229SL51/2022	10–2022	A	19.80	138.35	OP890338	PRJNA907066	SAMN32118114	SRR22580756
hRSV/A/USA/2022JECVB/2022	10–2022	A	19.58	79.68	OP890339	PRJNA907066	SAMN32118115	SRR22580755
hRSV/B/USA/2022YFP3F/2022	10–2022	B	19.09	111.2	OP890349	PRJNA907066	SAMN32118116	SRR22580754
hRSV/B/USA/2022M4SWF/2022	10–2022	B	18.65	103.76	OP890350	PRJNA907066	SAMN32118117	SRR22580753
hRSV/A/USA/2022AJXR3/2022	10–2022	A	18.58	95.86	OP890340	PRJNA907066	SAMN32118118	SRR22580752
hRSV/B/USA/202177756/2021	08–2021	B	19.4	355.53	OP965698	PRJNA907066	SAMN32118119	SRR22580750
hRSV/B/USA/202196775/2021	09–2021	B	22.9	19.97	OP965699	PRJNA907066	SAMN32118120	SRR22580749
hRSV/B/USA/202118974/2021	09–2021	B	21.1	140.21	OP965700	PRJNA907066	SAMN32118121	SRR22580748
hRSV/B/USA/202131818/2021	09–2021	B	22.5	43.97	OP965701	PRJNA907066	SAMN32118122	SRR22580747
hRSV/B/USA/202134981/2021	09–2021	B	23.9	238.93	OP965702	PRJNA907066	SAMN32118123	SRR22580746
hRSV/B/USA/202179926/2021	10–2021	B	20.4	350.92	OP965703	PRJNA907066	SAMN32118124	SRR22580745
hRSV/A/USA/202195752/2021	12–2021	A	18.6	115.71	OP965711	PRJNA907066	SAMN32118125	SRR22580744
hRSV/B/USA/202221067/2022	03–2022	B	21.6	40.88	OP965704	PRJNA907066	SAMN32118126	SRR22580743

Sequence name	Collection date	RSV subtype	Ct	Coverage†	Genbank no.	BioProject no.‡	BioSample no.‡	SRA fastq file‡
hRSV/B/USA/202210489/2022	02–2022	B	28.3	5,147.37	OP965708	PRJNA907066	SAMN32118130	SRR22580738
hRSV/B/USA/202275637/2022	02–2022	B	26.5	2,371.03	OP965709	PRJNA907066	SAMN32118131	SRR22580737
hRSV/B/USA/202188430/2021	12–2021	B	30	241.61	OP965710	PRJNA907066	SAMN32118132	SRR22580736
hRSV/B/USA/202194302/2021	08-2021	B	27.2	2,773.8	OP965705	PRJNA907066	SAMN32118127	SRR22580742
hRSV/B/USA/202114940/2021	09-2021	B	25.8	1,3972.6	OP965707	PRJNA907066	SAMN32118129	SRR22580739
hRSV/B/USA/202192941/2021	09-2021	B	30	2,652.03	OP965706	PRJNA907066	SAMN32118128	SRR22580741

*Ct, PCR cycle threshold; RSV, respiratory syncytial virus.
†Average depth of sequencing coverage (x-fold).
‡NCBI BioProject, BioSample, and SRA (sequence read archive) accession numbers (https://www.ncbi.nlm.nih.gov).

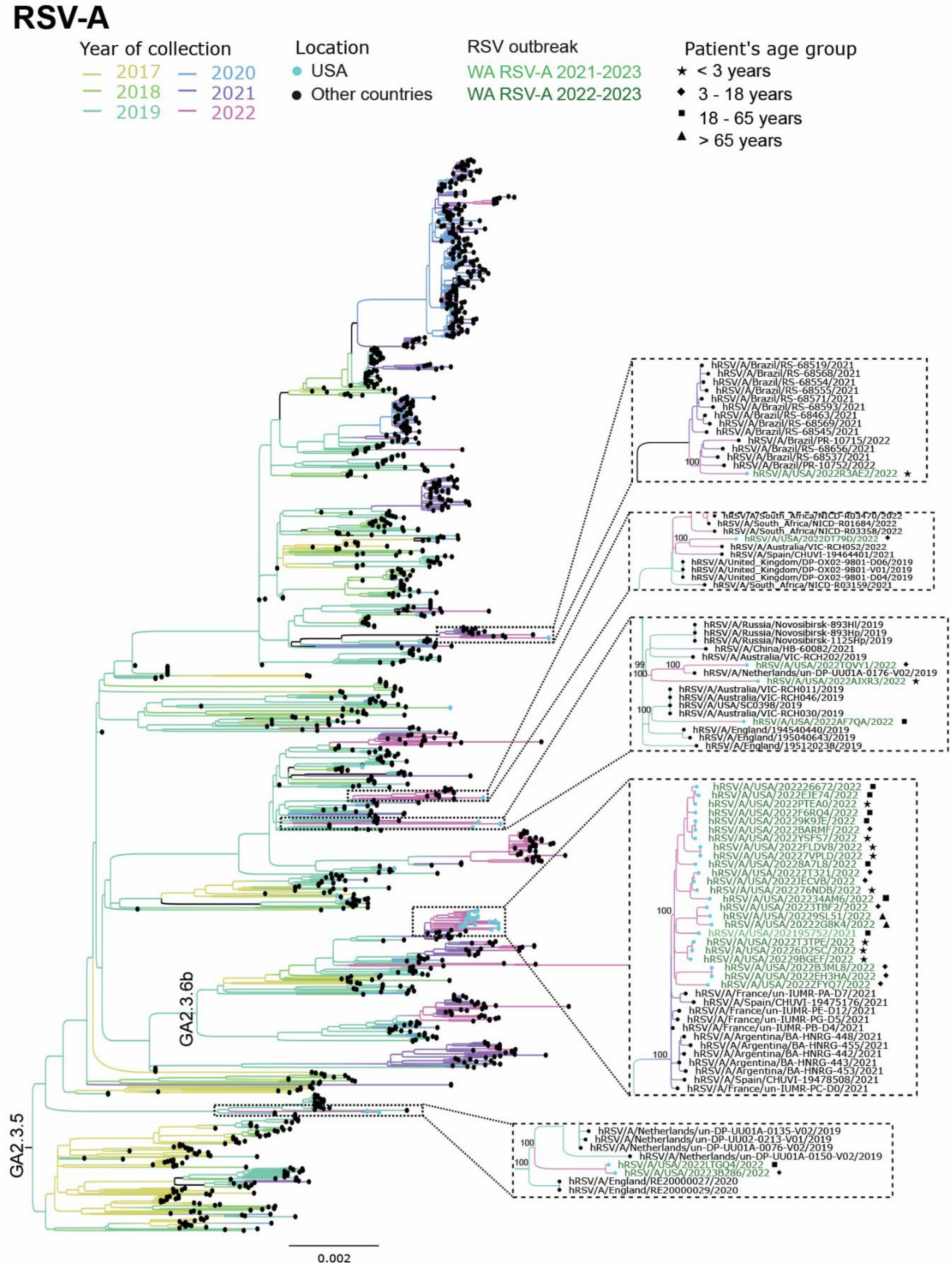

Appendix Figure 1. Complete phylogenetic analysis of respiratory syncytial virus, subtype A. Maximum-likelihood trees were constructed by using complete genomes of RSV-A (collected during 2017–2022) downloaded from GenBank and GISAID (https://www.gisaid.org) databases. Phylogenetic associations of

RSV-A genomes from our study are shown in boxes, which include bootstrap values for the main phylogenetic clades. Lineages GA2.3.5 and GA2.3.6b are labeled within their ancestral nodes. Collection years for specimens are depicted by tree branch color. RSV-A genomes from the United States are highlighted with light blue circles at branch tips. Washington RSV-A genomes from 2021–22 and 2022–23 outbreak seasons are highlighted in shades of green, and patient age groups are indicated by symbols. Scale bar indicates nucleotide substitutions per site. RSV-A, respiratory syncytial virus, subtype A.

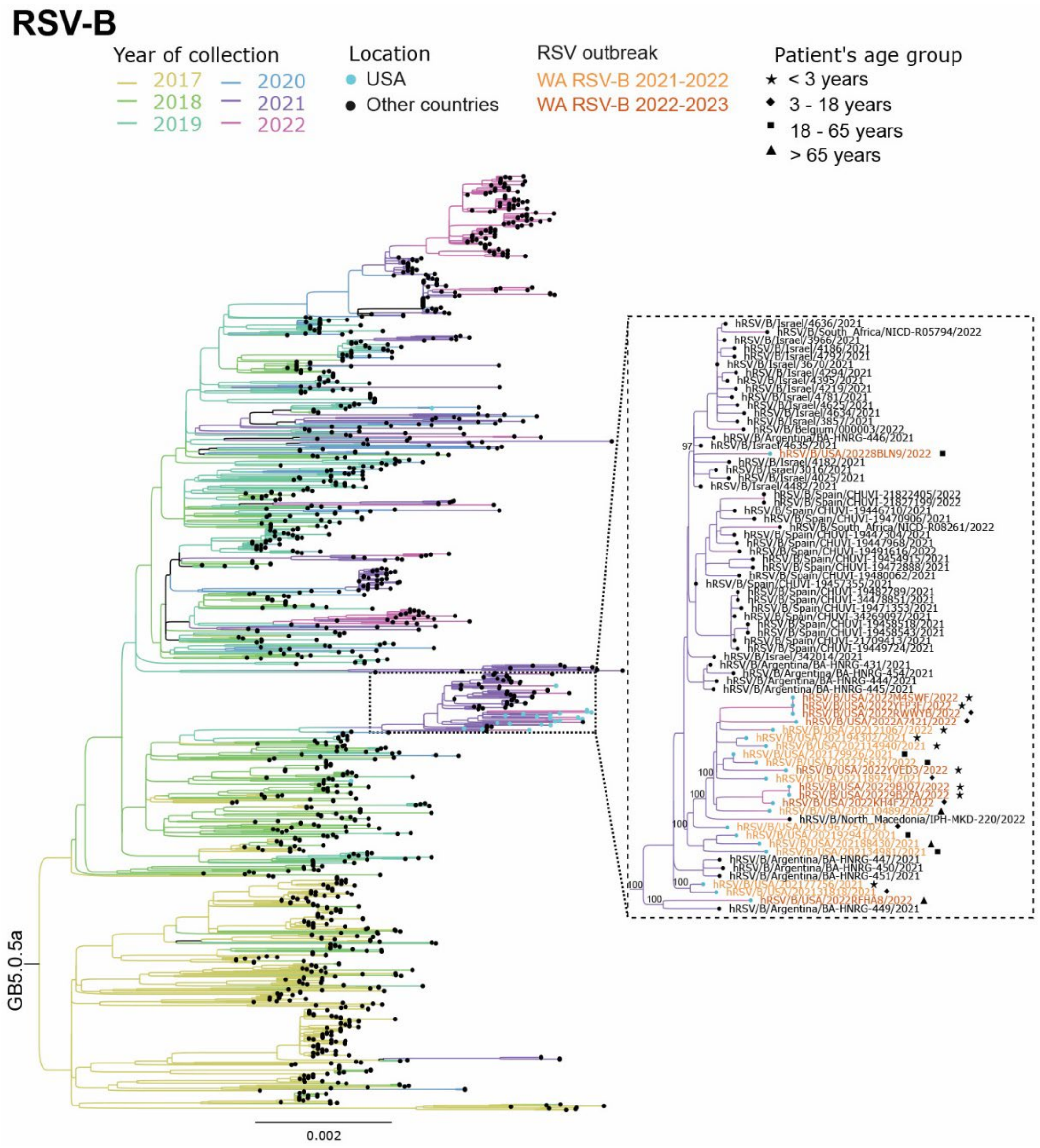

Appendix Figure 2. Complete phylogenetic analysis of respiratory syncytial virus, subtype B. Maximum-likelihood trees were constructed by using complete genomes of RSV-B (collected during 2017–2022) downloaded from GenBank and GISAID (https://www.gisaid.org) databases. Phylogenetic association of RSV-B genomes from our study are shown in the box inset, which includes bootstrap values for the main

phylogenetic clades. Lineage GB5.0.5a is labeled within its ancestral node. Collection years for specimens are depicted by tree branch color. RSV-B genomes from the United States are highlighted with light blue circles at branch tips. In the box inset, Washington RSV-B genomes from 2021–22 and 2022–23 outbreak seasons are highlighted in shades of orange, and patient age groups are indicated by symbols. Scale bar indicates nucleotide substitutions per site. RSV-B, respiratory syncytial virus, subtype B.
